# Alkaloid Biosynthesis in the Early Stages of the Germination of *Argemone mexicana* L. (Papaveraceae)

**DOI:** 10.3390/plants10102226

**Published:** 2021-10-19

**Authors:** Jorge Xool-Tamayo, Yahaira Tamayo-Ordoñez, Miriam Monforte-González, José Armando Muñoz-Sánchez, Felipe Vázquez-Flota

**Affiliations:** Centro de Investigación Científica de Yucatán, Unidad de Bioquímica y Biología Molecular de Plantas, Calle 43, No. 130 Chuburná, Mérida 97205, Mexico; jxool@cicese.mx (J.X.-T.); yahairatamayo@uadec.edu.mx (Y.T.-O.); mmg@cicy.mx (M.M.-G.); arms@cicy.mx (J.A.M.-S.)

**Keywords:** *Argemone mexicana*, berberine, benzylisoquinoline alkaloids, sanguinarine

## Abstract

The synthesis of the benzylisoquinoline alkaloids, sanguinarine and berberine, was monitored in *Argemone mexicana* L. (Papaveracea) throughout the early stages of its hypocotyl and seedling development. Sanguinarine was detected in the cotyledons right after hypocotyl emergence, and it increased continuously until the apical hook unbent, prior to the cotyledonary leaves unfolding, when it abruptly fell. In the cotyledonary leaves, it also remained at low levels. Throughout development, berberine accumulation required the formation of cotyledonary leaves, whereas it was quickly detected in the hypocotyl from the time it emerged. Interestingly, the alkaloids detected in the cotyledons could have been imported from hypocotyls, because no transcriptional activity was detected in there. However, after turning into cotyledonary leaves, important levels of gene expression were noted. Taken together, these results suggest that the patterns of alkaloid tissue distribution are established from very early development, and might require transport systems.

## 1. Introduction

**Figure 1:**
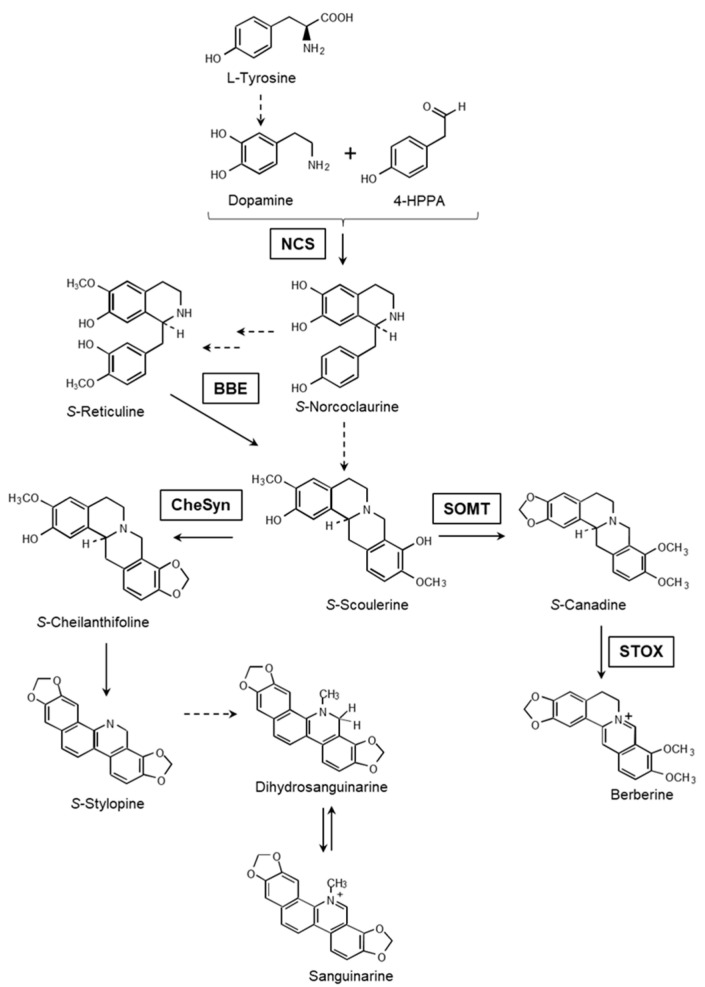
Condensed view of sanguinarine and berberine biosynthesis. The dashed lines represent multiple reactions. *NCS*, norcoclaurine synthase; *BBE*, berberine bridge enzyme; *SOMT*, *S*-scoulerine *O*-methyltransferase; *STOX*, tetrahydroprotoberberine oxidase; *CYP71914* (*ChESYN*), *S*-cheilanthifoline synthase.

Alkaloid synthesis is a complex process which is frequently associated with tissue and organ constitution. It can involve the participation of two or more organs, e.g., roots and aerial parts [1]; the development of specialized appendices and cell structures, such as trichomes and lactiferous cells [2,3]; and the formation of internal organelles, such as alkaloid accumulation vesicles [2]. Alkaloid synthesis in *Argemone mexicana* L. presents some of these requirements. The Argemone genus belongs to the Papaveraceae family. It includes over 25 species, all of them producers of the tyrosine-derived alkaloids known as benzylisoquinoline alkaloids (BIA’s) [4]. The main BIA’s identified in *A. mexicana* include the benzophenanthridine (sanguinarine and dihydrosanguinarine) and the protoberberine (berberine) groups [5] (Figure 1). In mature plants, these alkaloids present a clear tissue distribution, with sanguinarine being restricted to the roots and mature seeds, whereas berberine can be found in both aerial and underground parts, but it is absent in mature seeds [6]. Interestingly, the transcriptional activity related to the synthesis of these alkaloids in tissues of fully developed mature plants does not always match the presence of their products, suggesting long distance transport or communication between the above- and underground parts [6]. Both berberine and sanguinarine biosynthesis are activated from the early stages of seedling unfolding, and tissue spatial distribution is also set from these initial stages of development [7]. Interestingly, newly displayed cotyledons from developing seedlings have been able to accumulate important amounts of sanguinarine, although most of it was found in the seed coats. Because a limited biosynthetic capacity was detected in these tissues, this alkaloid was likely inherited from the original seed [7]. Sanguinarine increased markedly in seedlings later, upon the formation of the first pair of meristematic leaves, coinciding with the expression of biosynthetic genes [7]. This suggests that sanguinarine could be formed in the early developmental phases, contrasting with what occurs in mature plants [6,7]. It is noteworthy that sanguinarine synthesis in the aerial parts of *A. mexicana* was also detected in rootless in vitro shoots. However, once roots have formed, this organ turns into the only accumulation site [8,9]. On the other hand, berberine was mainly accumulated in the aerial parts of developing seedlings, regardless of the low expression of the biosynthetic genes in them, compared to that detected in radicles [7].

We are interested in establishing how early the actual onset of alkaloid synthesis occurs during the process of seedling germination in Argemone. Here, we followed the alkaloid synthesis throughout the very early stages of germination and organ development in *A. mexicana* hypocotyls. The data show that both berberine and sanguinarine synthesis in radicles is initiated once they emerge from seed cotyledons.

## 2. Results

Tissues of *A. mexicana* can accumulate both berberine and sanguinarine, depending on their morphogenic development. During early germination, alkaloid synthesis was associated to cotyledon display [7]. Here, we analyzed the previous events, i.e., radicle protrusion, hypocotyl development and apical hook formation. In order to confirm previous results, unfolding seedlings were also analyzed. Besides the alkaloid accumulation, the biosynthetic pathway was also monitored. This was achieved by estimating the transcript levels for *NCS* and *BBE*, which are involved in the early common reactions; *SOMT* and *STOX*, which are committed to berberine formation; and *ChESYN* (*CYP719A14*), which is involved in sanguinarine synthesis (Figure 1).

### 2.1. Hypocotyl and Seedling Development

**Figure 2:**
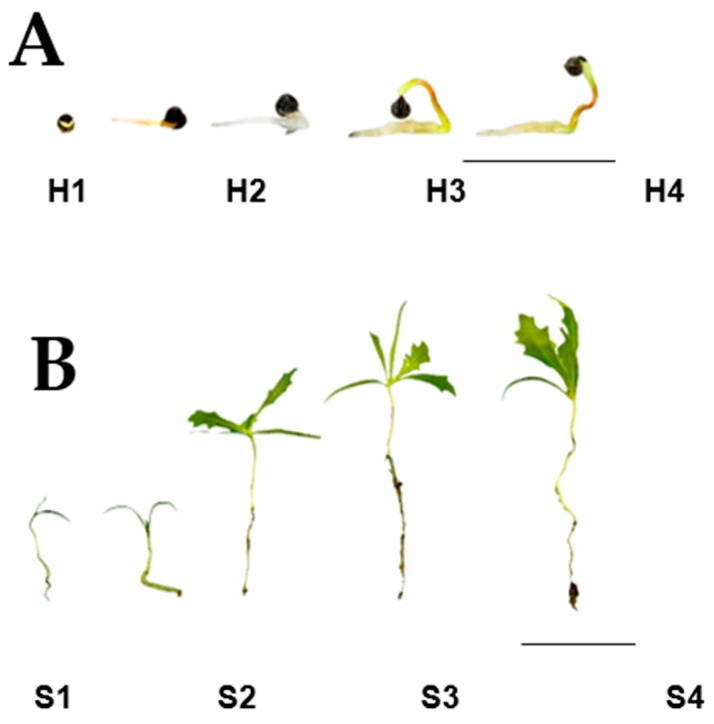
Aspects of *A. mexicana* hypocotyls (**A**) and seedlings (**B**). The scale bars represent 1 cm in each case.

Hypocotyls and seedlings were collected at different stages of development, registering the time, in days, after sowing. For this work, hypocotyls (stage H) were defined as those formed after radicle protrusion and before apical hook formation. Five developmental stages, named H1 to H5, were analyzed. H1 corresponded to seeds with cracked coats but prior to radicle protrusion; H2 and H3 were, respectively, those at radicle development and initial hypocotyl development, whereas H4 and H5 represented apical hook formation and hook straightening (Figure 2A). The seedling stage (stage S) was that starting with cotyledon unfolding, and it was also followed through five stages: S1 to S5. S1 and S2 corresponded to the cotyledonary leaves up to the detection of apical meristem sprouting; S3 and S4 corresponded to the expansion of the first pair of true leaves, whereas the formation of the second pair corresponded to S5 (Figure 2B).

### 2.2. Alkaloid Synthesis at the Hypocotyl Stage

**Figure 3:**
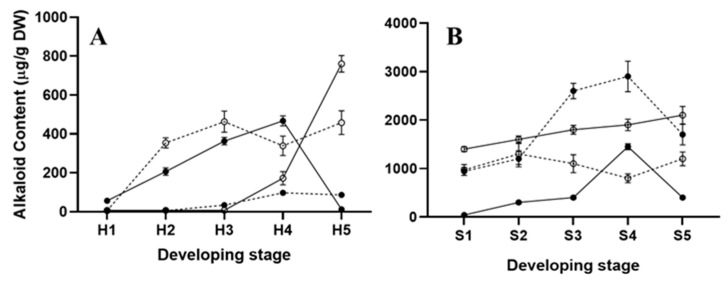
Alkaloid contents in different tissues in the hypocotyl (**A**) and seedling (**B**) throughout different developmental stages in *A. mexicana*. The developmental stages labeled 1 to 5 correspond to H1 to H5 (**A**) or S1 to S5 (**B**), as described in the text. The closed and open circles represent the alkaloid contents in cotyledons and radicles, respectively, whereas the continuous and dashed lines stand for sanguinarine and berberine, respectively. The average of triplicates with the standard deviation is given.

In mature seeds, prior to germination, sanguinarine is accumulated in high amounts (ca. 1200 µg/g DW^−1^), mainly in the seed coats, where it remains during seedling emergence [7]. The alkaloid amounts recovered from uncoated seed cotyledons represent a minor proportion of the seed’s total alkaloid content [7]. Hence, because the seed coats were removed before analysis, the contents detected in cotyledons from hypocotyls represented the newly formed alkaloids. The sanguinarine contents in cotyledons increased continuously through stages H2 to H4 (Figure 2A) to a maximum (near to 550 µg/g DW^−1^), shortly before hook straightening (Figure 3A). These maximal sanguinarine values represented ca. 50% of those found in the complete seeds, prior to germination [7], and they occurred once the apical hook was well defined (H4; Figure 2A). It is noteworthy that sanguinarine markedly decreased when the cotyledons straightened, just before the cotyledonary leaves’ emergence (H5; Figure 2A). Hook straightening resulted in the loss of the sanguinarine accumulation in these parts (Figure 2A and Figure 3A). In radicles, sanguinarine could be barely detected until stage H4, reaching its maximum before the cotyledon leaves were displayed at H5 stage (Figure 2A and Figure 3A). The berberine contents in the cotyledons at the hypocotyl stage remained at low levels (<80 µg/g DW^−1^), and were detected at the beginning of hook formation (H4 and onwards; Figure 3A). Contrastingly, considerable amounts (ca. 400 µg/g DW^−1^) were observed in the radicles, as soon as they emerged (H2), and remained in such values up to the hook straightening (H5; Figure 3A).

**Figure 4:**
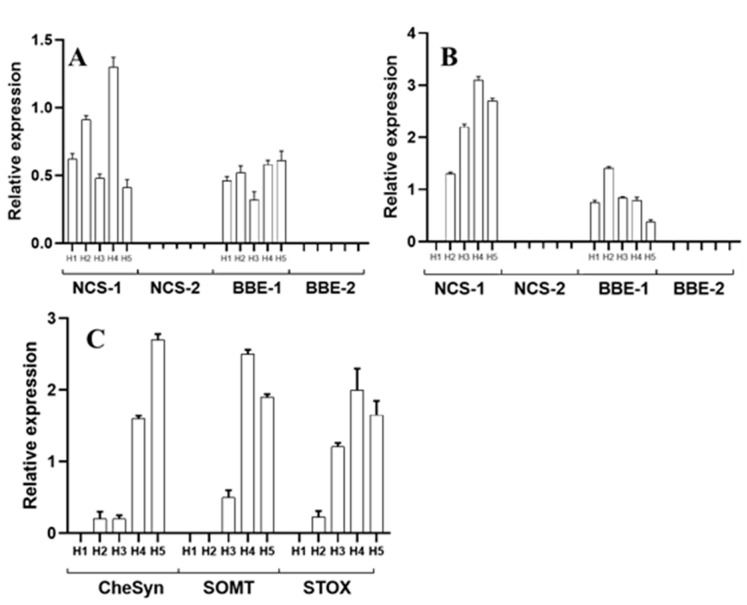
Relative abundance of the transcript involved in alkaloid biosynthesis in *A. mexicana* hypocotyls. The developmental stages correspond to H1 to H5, as shown in Figure 2A. (**A**,**B**) are the relative transcript abundance for *NCS-1* and *BBE-1* in the cotyledons and radicles, respectively. *NCS-2* and *BBE-2* were below the detection limits. (**C**) is the relative transcript abundance for *SOMT*, *STOX* and *CYP71914* (*ChESYN*) in the radicles (the levels in cotyledons were undetectable). The average of triplicates with the standard deviation is given. *ACTIN* was used as the reference gene.

Transcriptional activity related to alkaloid biosynthesis was detected in both the hypocotyls’ cotyledons and radicles (Figure 4). Markers associated to the early reactions (*NCS* and *BBE*) were detected as a single isoform, equally in cotyledons and radicles (*NCS-1* and *BB-1*; Figure 4A,B). However, differences were noticed in markers corresponding to specific alkaloids, because neither those associated to sanguinarine (*ChESYN*) nor berberine (*SOMT* and *STOX*) were observed in cotyledons. In contrast, both of them could be detected in radicles (Figure 4C). In cotyledons, *NCS-1* and *ChESYN* transcripts followed the same trend as the sanguinarine accumulation, i.e., a continuous increase throughout the different developmental stages, up to hook formation (H4), followed by an abrupt decrease after straightening (H5) (Figure 2A). Conversely, *BBE-1* was maintained at similar levels from the very early stages through to H5. These results suggest that the sanguinarine accumulated in the cotyledons could have been imported from developing hypocotyls. Correspondently to the lack of significant amounts of berberine in the cotyledons (Figure 3A), neither the expression of *SOMT* nor of *STOX* were detected in this part. In hypocotyls, the expression of only the *NCS-1* isoform was observed, and it increased as the development proceeded (Figure 4B). Interestingly, a relatively high expression was detected at the medium stages (H2 and H3, corresponding to the early and late hook formation; Figure 2A), and seemed related to berberine synthesis, because no sanguinarine was noticed in the radicles at such periods (Figure 3A). *ChESYN* was also barely detected at the early developmental stages in hypocotyls, showing a noticeable increase simultaneously with the sanguinarine accumulation (Figure 3A and Figure 4C). Although it was at low levels, the expression of *SOMT* and *STOX* (Figure 4C) coincided with the presence of the alkaloids. This suggests that, as a difference to cotyledons, the alkaloids detected in hypocotyls could have been formed in the same tissue.

### 2.3. Alkaloid Synthesis at the Seedling Stage

The alkaloid amounts in the seedlings were augmented notably once the cotyledonary leaves were open (Figure 3B). However, low sanguinarine amounts were observed, except at the S4 stage, when the formation of the second pair of meristematic leaves occurred (Figure 2B and Figure 3B). In the radicles, sanguinarine presented a minor increase from the amounts detected prior to the cotyledon opening, and remained between 1400 and 2000 µg/g DW^−1^ throughout the analyzed period (Figure 3B). In contrast, the transition from the H to S stage produced a marked increase in berberine accumulation (from less than 80 up to 1000 µg/gDW^−1^ between the H5 and S1 stages; Figure 2B and Figure 3B). The maximal values were observed once the meristematic leaves were formed (S3 and S4; around 3000 µg/gDW^−1^) and, although it decreased afterwards, it was kept above 1400 µg/gDW^−1^ (Figure 3B). In the radicles, the berberine accumulation followed a similar trend to that of sanguinarine, showing little variation throughout development (ca. 1000 µg/g DW^−1^; Figure 3B). In this way, berberine was the main alkaloid in the aerial parts once they were formed.

**Figure 5:**
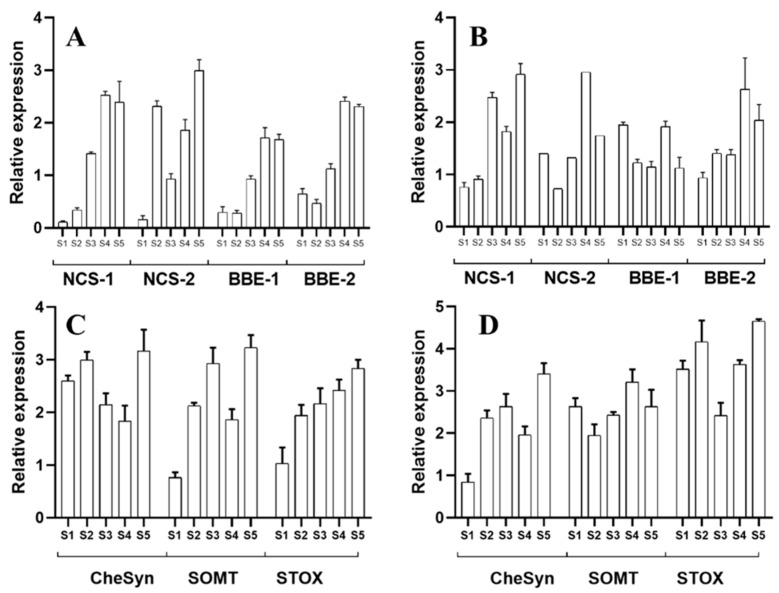
Relative abundance of the transcript involved in alkaloid biosynthesis in *A. mexicana* hypocotyls. The developmental stages correspond to S1 to S5, as shown in Figure 2B. (**A**,**B**) are the relative transcript abundance for *NCS* and *BBE* in cotyledons and radicles, respectively. (**C**,**D**) are the relative transcript abundance for *SOMT*, *STOX* and *CYP71914* (*ChESYN*). The average of triplicates with the standard deviation is given. *ACTIN* was used as the reference gene.

Contrasting with the hypocotyls, the transcriptional activity related to alkaloid synthesis was equally detected in both the aerial and underground parts of the seedlings. Moreover, the two isoforms for *NCS* and *BBE* were equally detected in the aerial parts and radicles, although no differential expression patters were noticed for any of these isoforms. As a general trend, a developmentally related increase in expression was noticed for transcripts corresponding to both enzymes (Figure 5A,B). Interestingly, *ChESYN* expression was observed in the cotyledonary leaves, even when no significant accumulation of sanguinarine was detected in these parts (Figure 5C). The expression levels were similar to those in the radicles (Figure 5D), which presented much higher sanguinarine amounts (Figure 3B). These data suggest that the cotyledonary leaves could contribute to the sanguinarine levels found in the radicles. Contrastingly, the expression of berberine-formation-related genes (*SOMT* and *STOX*) was detected in high levels throughout the analyzed developmental stages in both the aerial parts (Figure 5C) and radicles (Figure 5D), with a good correspondence to the alkaloid amounts detected in these tissues.

## 3. Discussion

Germination and hypocotyl/seedling development represent the interpretation of the plant’s master plan imprinted in the embryo. Bipolar embryo development sets the patterns for aerial and underground organ formation [10,11]. In natural conditions, the early stages of seed germination take place underground, resulting in hypocotyls with an apical hook and closed cotyledons. Once hypocotyls emerge from the soil, light triggers hook straightening and the displaying of green cotyledons, leading to a switch from heterotrophy to autotrophy. Such a modification involves both morphological and metabolic changes [12], including the onset of secondary metabolism [13].

The alkaloid synthesis in *A. mexicana* occurred from the very early stages of seedling germination, once the hypocotyls protruded (Figure 2, Figure 3, Figure 4 and Figure 5). In the very early phases, the cotyledons from hypocotyls accumulated sanguinarine in higher amounts than the radicles (Figure 3A), and this coincided with the transcriptional activity detected in radicles (Figure 4). The sanguinarine in the radicles was augmented only when the cotyledons were ready to open, and kept on increasing during the aerial part development (Figure 3A,B). This suggests the operation of transport systems, moving alkaloids from the hypocotyl radicles to the cotyledons. Interestingly, the aerial parts started expressing *ChESYN* even when no sanguinarine was accumulated in them (with the exception of the S4 peak). Taken together, these results suggest that, in the very early stages of germination, radicles might carry out most of the sanguinarine synthesis for exportation to the cotyledons. However, once the cotyledons expanded, radicles seem to store the formed sanguinarine, in addition to some provided from the aerial parts. This does not seem to be the case for berberine, which coincided both in the cotyledons and radicles with the *SOMT* transcripts (Figure 2, Figure 3, Figure 4 and Figure 5). Interestingly, during the early stages of development, prior to the meristematic leaves’ formation, no vascular tissues were formed [13]. This suggests that alkaloids could move between tissues using a cell-to-cell transport mechanism. Such a type of mechanism has been described for other alkaloids, such as the pirrolizidines [14]. In fact, a transcriptome from developing seedlings of *A. mexicana* [6,7] revealed the presence of several putative ABC proteins, which have been involved in benzylisoquinoline alkaloid transport [15].

## 4. Materials and Methods

### 4.1. Plant Materials

The seed coats were mechanically detached from hypocotyls for the alkaloid analysis. The samples were freeze-dried and ground to a fine powder using a mechanical mill (IKA Tube Mill CS001; Wilmington NC). The alkaloids were extracted from 50 mg powdered tissues in 10 mL 0.5% hydrochloric acid in methanol, as previously described [16]. Briefly, the powders were incubated for two hours with gentle shaking at room temperature, and the debris was separated by filtration. Next, 1 mL aliquots were centrifuged for the further remotion of the tissue remains, and were used for the chromatographic analysis. The alkaloids were separated by TLC, using a mixture of n-butanol:water:NH_4_OH (8:1:1), or benzene:ethanol (9:1), for berberine (*R_f_* 0.29) and sanguinarine (*R_f_* 0.53) detection, respectively. After the chromatographic separation, the alkaloids were quantified by in situ fluorescence in a Camag TLC Scanner 4 (Muttenz Switzerland) controlled by the WinCATS 1.4.10 planar chromatography manager [16]. The analysis was performed in triplicates.

### 4.2. Nucleic Acid Analysis

The total RNA was extracted as described elsewhere [17]. The analyzed genes corresponded to common biosynthetic reactions, as well as being committed to the specific synthesis of berberine and sanguinarine (Figure 1). Norcoclaurine synthase (*NCS-1* and *-2*; EU881891 and EU881893), the berberine bridge enzyme (*BBE-1* and *-2*; EU881889 and EU881890), scoulerine *O*-methyltransferase (*SOMT*; KT984756), *STOX* (tetrahydroprotoberberine oxidase; HQ116698) and *S*-cheilanthifoline synthase (*ChESYN*; *CYP719A14*; EF451152) were analyzed to monitor the alkaloid biosynthetic pathways (Figure 1). *NCS* and *BBE* represent the early and common biosynthetic reactions; *SOMT* and *STOX* represent those committed to berberine, whereas *ChESYN* is specific to sanguinarine formation [7,18,19]. Specific oligonucleotide primers [7] were used to analyze the transcripts’ relative abundance by quantitative real time PCR (qRT-PCR). See Appendix A for the primer sequences. The first strand cDNA was synthesized using M-MLV reverse transcriptase (Invitrogene, Carlsbad, CA, USA) according to standard procedures. The qRT-PCR was performed using an Eco^Tm^ Real Time PCR System (Illumina, San Diego, CA, USA). The reaction mixture (20 µL) contained 10 μL SYBR Green Mastermix (Applied Biosystems, United Kingdom), 10 µM of each primer, and 5 ng of the RT products. A 35-cycle program was used (50, 30, and 30 s at 95, 60, and 60 °C for the DNA denaturation, primer alignment and amplification, respectively). The gene transcripts were quantified using the cycle threshold value (Ct), using *ACTIN* as a reference [20]. Prior to performing the qPCR, amplicon’s identity was verified by sequencing it after the standard PCR with the same set of primers. The analysis was performed in triplicates.

## 5. Conclusions

Taken together, these results suggest that the patterns of alkaloid tissue distribution are established from very early development, and might require transport systems.

## Data Availability

All of the data are publicly available in the databases mentioned.

## Appendix A

The specific primers were taken from [6,7]. Accession numbers: *NCS-1* and *-2* (norcoclaurine synthase), EU881891 and EU881893, respectively; *BBE-1* and *-2* (berberine bridge enzyme), EU881889 and EU881890, respectively; SOMT (scoulerine *O*-methyltransferase), KT984756; *STOX* (tetrahydroprotoberberine oxidase), HQ116698; and *ChESYN/CYP719A14* (cheilanthifoline synthase), EF451152.

**Table A1:** Sequence of the primers used for qRT-PCR.

Gene	Direction ^1^	Sequence (5′–3′)
*ACTIN*	Fwd	CACIACTACTGCTAAACGGGAAA
	Rvs	ACATCTGCTGGAAGGTGCTG
*TyDC*	Fwd	GTTGAACCAGGTTATTTACG
	Rvs	CGTATTCTTTCGCAACCTC
*NCS-1*	Fwd	CATCGCTAATTACGTTCTCAAGAATCA
	Rvs	ATAGTAGTACATGGAATTACCTGGATGGGA
*NCS-2*	Fwd	CGTACCATTGAAATCCATGTCAGAA
	Rvs	CATCGGACGGTAATTACCCATG
*BBE-1*	Fwd	CATCTTTGTTCATCATCATCTTCTTCTTCTTCTT
	Rvs	GATCCTCTTGTGCAACATCTAACGGT
*BBE-2*	Fwd	CTCATCTTTGTTCATCTTCTTTTCTGTGC
	Rvs	GATCCTCTTGTGCAACATCTAACGGT
*CHESYN*	Fwd	AGGTCTTCAAGGTGTTGCCC
*(CYP719A14)*	Rvs	TCTTTTCCCGCCCGTAACAT
*SOMT*	Fwd	ATCCTATCCATGTCTACGAGGGCTATT
	Rvs	CCAGTACCACCACCAACATCTAACA
*STOX*	Fwd	GGTTAGGAAATATGGACTTG
	Rvs	ATAACATTGCTGGTGAATCT

^1^ Fwd, forward; Rvs, reverse.
